# Supplementary material for: Mechanisms governing the pioneering and redistribution capabilities of the non-classical pioneer PU.1
Source: Nat Commun. 2020 Jan 21;11:402. doi: 10.1038/s41467-019-13960-2 (PMC6972792; doi:10.1038/s41467-019-13960-2)
Supplement: Supplementary file 7 — Source data [file 41467_2019_13960_MOESM7_ESM.zip › Source_Data/Figure6/Figure6B_MotifScanOutput/ETS1_PU.1/homerResults/motif18.similar.html]

motif18

## Information for motif18

G
A
T
C
T
G
A
C
A
G
T
C
C
A
G
T
A
G
T
C
A
G
T
C
A
G
T
C
G
T
A
C
A
G
T
C
C
G
T
A
A
G
T
C
  
Reverse Opposite:  

T
C
A
G
G
C
A
T
A
C
T
G
A
C
T
G
A
C
T
G
A
C
T
G
C
T
A
G
G
T
C
A
C
T
A
G
A
C
T
G
C
T
A
G
  

|  |  |
| --- | --- |
| p-value: | 1e-127 |
| log p-value: | -2.927e+02 |
| Information Content per bp: | 1.689 |
| Number of Target Sequences with motif | 2490.0 |
| Percentage of Target Sequences with motif | 15.73% |
| Number of Background Sequences with motif | 2665.4 |
| Percentage of Background Sequences with motif | 8.31% |
| Average Position of motif in Targets | 101.4 +/- 54.0bp |
| Average Position of motif in Background | 99.8 +/- 63.7bp |
| Strand Bias (log2 ratio + to - strand density) | 0.0 |
| Multiplicity (# of sites on avg that occur together) | 1.12 |
| Motif File: | file (matrix) reverse opposite |

### Similar de novo motifs found

|  |  |  |  |  |  |  |  |
| --- | --- | --- | --- | --- | --- | --- | --- |
| Rank | Match Score | Redundant Motif | P-value | log P-value | % of Targets | % of Background | Motif file |
| 1 | 0.827 | C G A T G T C A T A G C A C G T G A T C A T G C A G T C A G T C G T A C | 1e-115 | -266.918254 | 11.88% | 5.74% | motif file (matrix) |
| 2 | 0.814 | T G A C A G T C A T G C T A G C T A G C T G A C G T A C | 1e-78 | -181.705701 | 26.85% | 19.20% | motif file (matrix) |
| 3 | 0.851 | G C T A T C A G A C T G C T A G A T C G T A C G G T A C C T A G A T C G C T A G T C A G T A G C T G C A C T A G | 1e-73 | -169.900818 | 9.96% | 5.36% | motif file (matrix) |
| 4 | 0.839 | A C T G A C T G A C T G A T C G A T C G T C A G A C T G A C T G | 1e-68 | -157.418187 | 16.60% | 10.82% | motif file (matrix) |
| 5 | 0.761 | A G C T C A T G T A C G A G T C G A T C T G A C G T A C C A T G T A G C G T A C G A T C G T C A A T G C | 1e-55 | -128.755134 | 6.87% | 3.54% | motif file (matrix) |
| 6 | 0.814 | G C T A C A T G G A C T G A C T A T G C A G T C A C G T G A T C A G T C A G T C G A T C G A T C | 1e-45 | -103.708655 | 3.94% | 1.73% | motif file (matrix) |
